# Usp5 functions as an oncogene for stimulating tumorigenesis in hepatocellular carcinoma

**DOI:** 10.18632/oncotarget.16901

**Published:** 2017-04-06

**Authors:** Yi Liu, Wei-Mao Wang, Ying-Fei Lu, Lu Feng, Li Li, Ming-Zhu Pan, Yu Sun, Chun-Wai Suen, Wei Guo, Jian-Xin Pang, Jin-Fang Zhang, Wei-Ming Fu

**Affiliations:** ^1^ Guangdong Key Laboratory for Research and Development of Natural Drugs, Guangdong Medical University, Zhanjiang, Guangdong, China; ^2^ School of Biomedical Sciences, Faculty of Medicine, The Chinese University of Hong Kong, Hong Kong, China; ^3^ Department of Orthopaedics and Traumatology, The Chinese University of Hong Kong, Prince of Wales Hospital, Shatin, Hong Kong, China; ^4^ Shenzhen Enhance Techology Co. Ltd., Shenzhen, Guangdong, China; ^5^ Shenzhen Ritzcon Biological Techology Co. Ltd., Shenzhen, Guangdong, China; ^6^ School of Pharmaceutical Sciences, Southern Medical University, Guangzhou, Guangdong, China; ^7^ Guangzhou Institute of Advanced Technology, Chinese Academy of Sciences, Guangzhou, Guangdong, China

**Keywords:** Usp5, epatocellular carcinoma, proliferation, migration, P14

## Abstract

As deubiquitinases, several ubiquitin specific protease members have been reported to mediate tumorigenesis. Although ubiquitin specific protease 5 (Usp5) was previously demonstrated to suppress p53 transcriptional activity and DNA repair, its role in carcinogenesis remains elusive. In this study, we sought to define a novel role of Usp5 in tumorigenesis. It was found that Usp5 was significantly upregulated in hepatocellular carcinoma (HCC) cells and most clinical specimens. Further functional investigation also showed that Usp5 knockdown suppressed cell proliferation, migration, drug resistance and induced apoptosis; on the other hand, Usp5 overexpression promoted colony formation, migration, drug resistance and tumorigenesis. Additionally, the inactivated p14^ARF^-p53 signaling was observed in Usp5 overexpressed HCC cells, while this signaling was activated by Usp5 knockdown. Therefore, our data demonstrated that Usp5 contributed to hepatocarcinogenesis by acting as an oncogene, which provides new insights into the pathogenesis of HCC and explores a promising molecular target for HCC diagnosis and therapy.

## INTRODUCTION

Hepatocellular carcinoma (HCC), the most common primary malignant liver cancer is a global public health problem that accounts for approximately 500,000 deaths annually. Although advances in HCC diagnosis and treatment have increased the possibility of cure, HCC remains largely incurable because of poor prognosis and recurrence [1, 2]. Therefore, the development of innovative and targeted therapies is imperative and of high clinical significance to HCC patients.

Ubiquitination is an important post-translational modification to regulate most cellular process from DNA transcription to protein degradation. This process is mediated by deubiquitinating enzymes (DUBs) through ubiquitin-proteasome system (UPS) [3–5], which play key roles in many activities such as cell cycle [6], apoptosis [7–10], receptor signaling and endocytosis [11–12]. The increasing evidences demonstrate multiple UPS-involved regulation mechanisms in the pathogenesis of HCC [13–14], suggesting that it may be developed as a novel therapy for this devastating disease.

As a member of the ubiquitin specific protease (Usp) family of DUBs, Usp5 was reported as an exopeptidase that hydrolyzes isopeptide bonds in poly-ubiquitin from their free carboxy-terminal ends to produce mono-ubiquitin [15–17]. Recently, it has been shown that Usp5 knockdown induces p53 activation [18], and Usp5 controls activation of apoptotic signaling as well as Jun N-Terminal kinase pathway during eye development [19]. However, the role of Usp5 in tumorigenesis remains elusive. In the present study, upregulation of Usp5 was found in a panel of HCC cell lines and most clinical specimens. Further investigations discovered that Usp5 acts as a novel oncogene and its overexpression promotes the viability, migration, drug resistance and tumorigenicity *via* alleviating p14^ARF^-p53 signaling, which contributes to the tumorigenesis of HCC.

## RESULTS

### Usp5 was upregulated in human HCC cell lines and most clinical specimens

**Figure 1:**
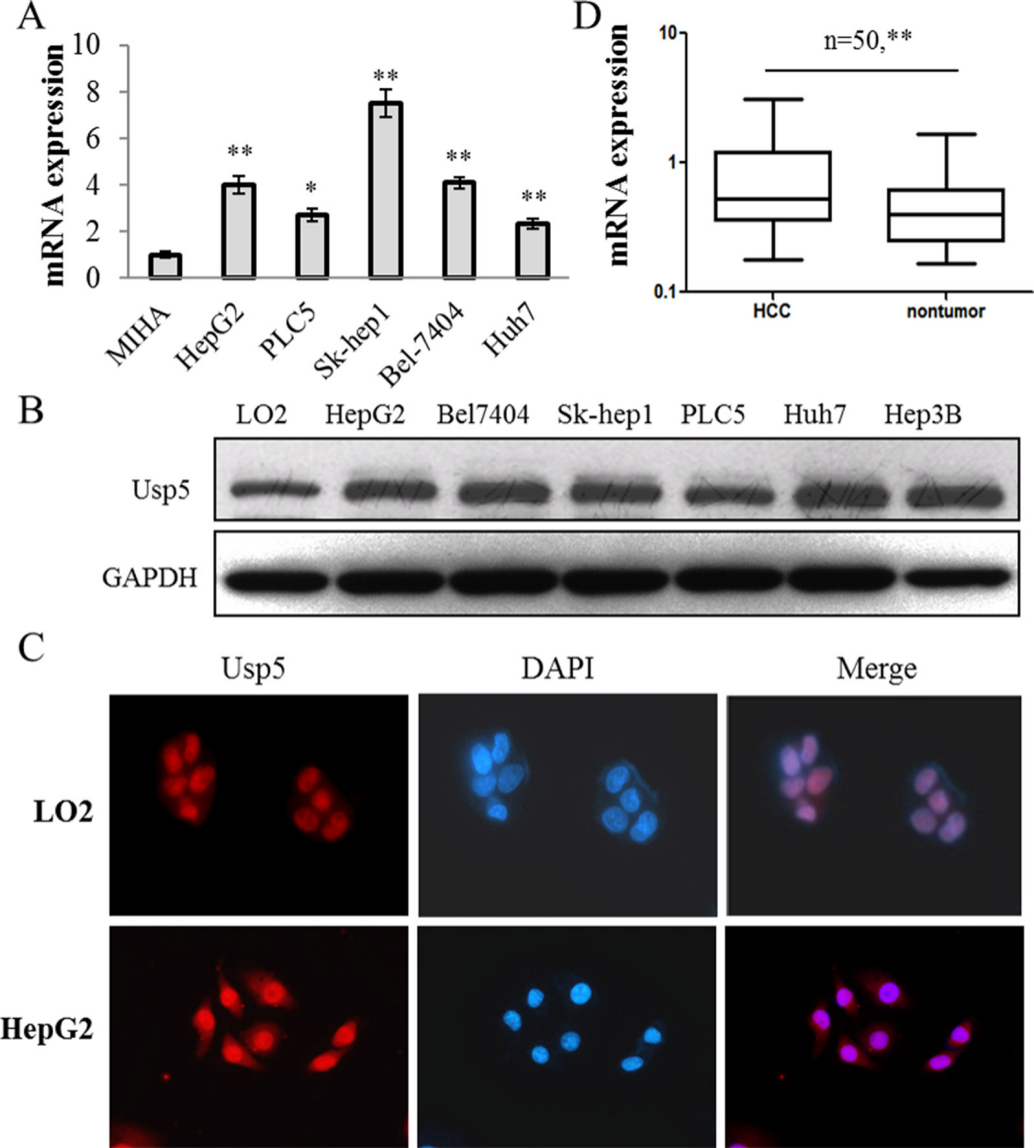
Usp5 was significantly upregulated in HCC cells and most clinical specimens (**A** and **B**) Usp5 was upregulated in a panel of HCC cells at mRNA (A) and protein levels (B). (**C**) Usp5 was localized in nuclei and its expression was increased in HCC cells compared with MIHA cells by using fluorescence immunocytochemistry analyses. (**D**) Usp5 was upregulated in 50 paired HCC clinical specimens. *P < 0.05; **P < 0.01.

Previous study has shown that Usp5 knockdown promoted p53 activation, so we suppose that Usp5 may be involved in carcinogenesis. In the present study, it was found that Usp5 was significantly upregulated in most HCC cells at mRNA and protein levels (Figure 1A and 1B). Fluorescence immunocytochemistry analysis showed an obvious enrichment of nuclear Usp5 in HepG2 cells (Figure 1C). Furthermore, we also found that Usp5 expression was increased in HCC tissues compared to their adjacent non-tumor tissues (Figure 1D). Therefore, upregulation of Usp5 is a frequent event in human HCC, indicating that Usp5 may be involved in malignant tumor development and progression.

### Usp5 knockdown suppressed cell viability and induced apoptosis in HCC cells

**Figure 2:**
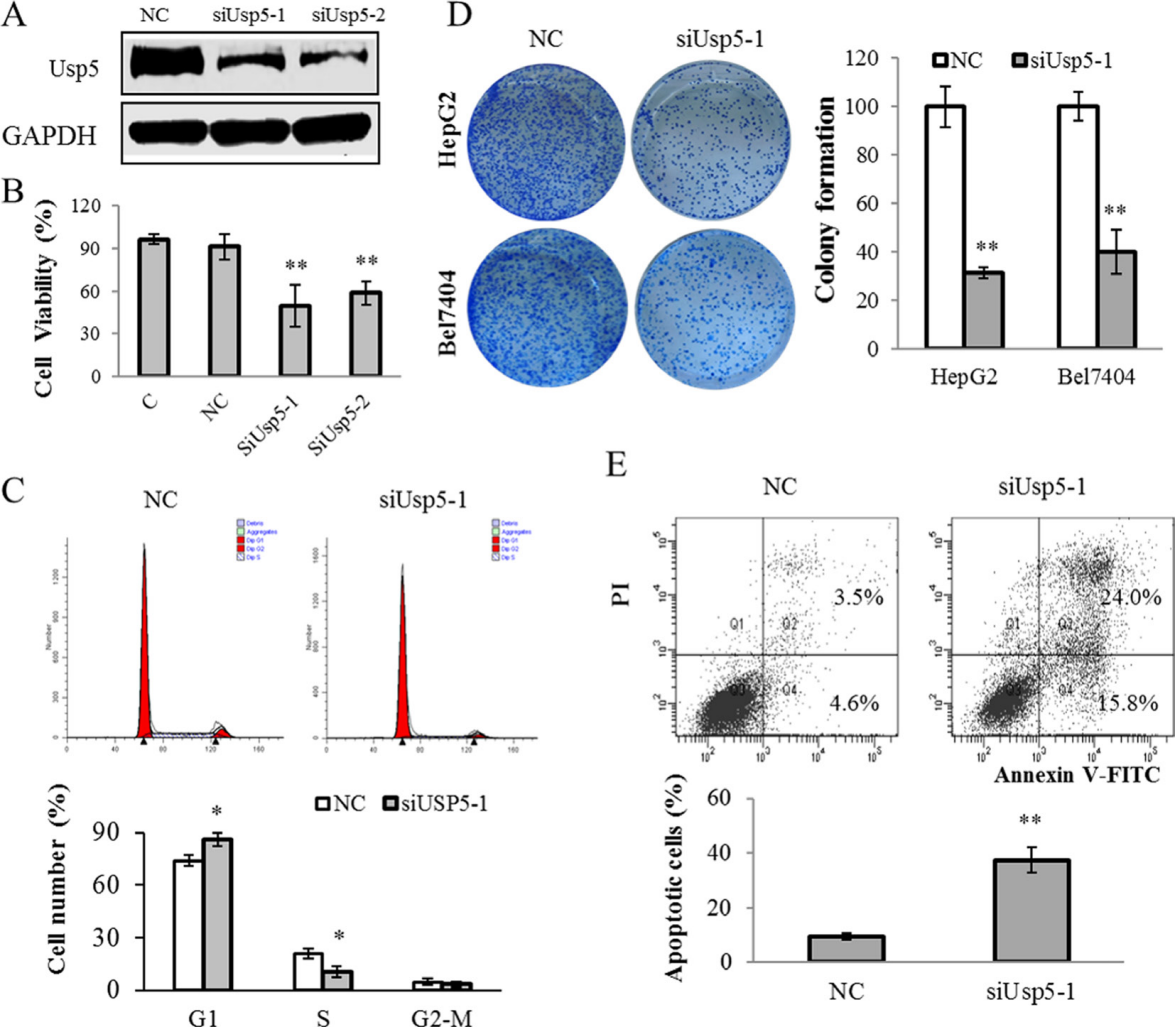
Usp5 knockdown suppressed cell growth and induced apoptosis in HepG2 cells (**A**) Usp5 expression was suppressed by siUsp5-1 and siUsp5-2 in HepG2 cells. (**B** and **C)** siUsp5 suppressed the cell viability at day 3 (B) and induced the G1-phase arrest in HepG2 cells (C). (**D**) the representative images and statistical analyses of colony formation assays in siUsp5 transfected HCC cells. (**E**) siUsp5 induced more apoptotic cells of HepG2 cells by flow cytometry analyses. Top, results of one such assay; and bottom, mean ± SD of three independent experiments. *P < 0.05; **P < 0.01.

To further validate the function of Usp5 in hepatocarcinogenesis, we silenced Usp5 by using its specific siRNAs (siUsp5) and the results showed that the expression of Usp5 was significantly reduced by siUsp5-1 and siUsp-2 (Figure 2A). The siUsp5-1 was selected to be applied to the following experiments. HepG2 cells were transfected with siUsp5-1, and a dramatically suppressive effect of siUsp5 on cell viability was observed in HCC cells at day 3 (Figure 2B). Moreover, the cell cycle distribution demonstrated that siUsp5-1 induced an increased HepG2 cell percentage in G1-phase and a decreased percentage in S-phase (Figure 2C), indicating a growth-suppressive effect resulted from G1-phase arrest. Consistent with these results, siUsp5-1 also induced cell cycle arrest in Bel7404 cells (Supplementary Figure 1). Furthermore, the capacity of colony formation was evaluated and results showed that siUsp5-1 transfected HCC cells displayed much fewer and smaller colonies compared with those obtained with NC transfected cells (Figure 2D). For the apoptosis assays, HepG2 cells were transfected with siUsp5-1, and more apoptotic cells were observed in this transfected cells (Figure 2E).

### Usp5 overexpression promoted cell growth *in vitro* and stimulated tumorigenicity *in vivo*

**Figure 3:**
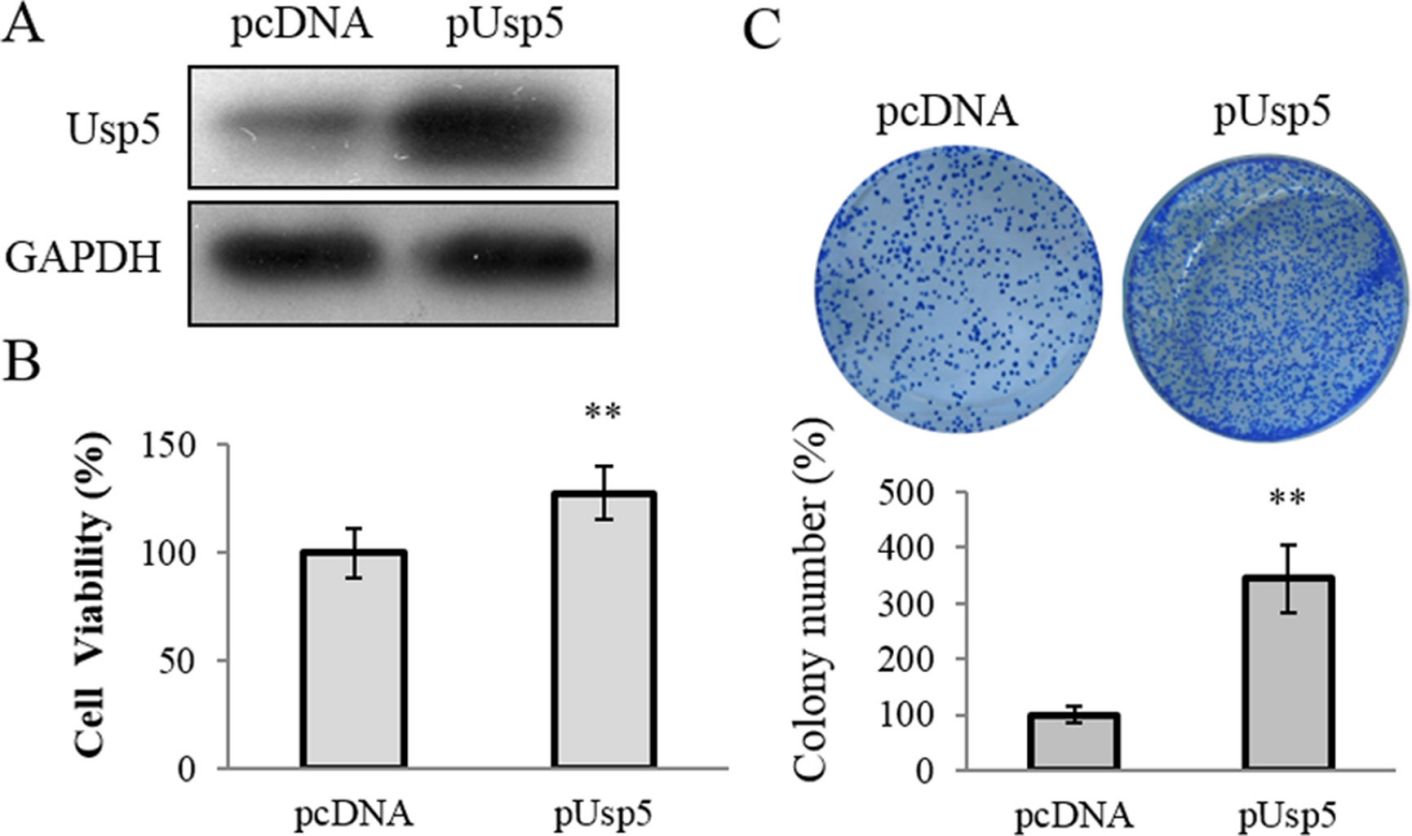
Usp5 overexpression promoted cell proliferation in HepG2 cells (**A**) Usp5 expression was significantly increased in the pUsp5 transfected HepG2 cells. (**B**) Usp5 overexpression promoted the cell viability in HepG2 cells. (**C**) the representative images and statistical analyses of colony formation assay of pUsp5 transfected HepG2 cells. **P < 0.01.

**Figure 4:**
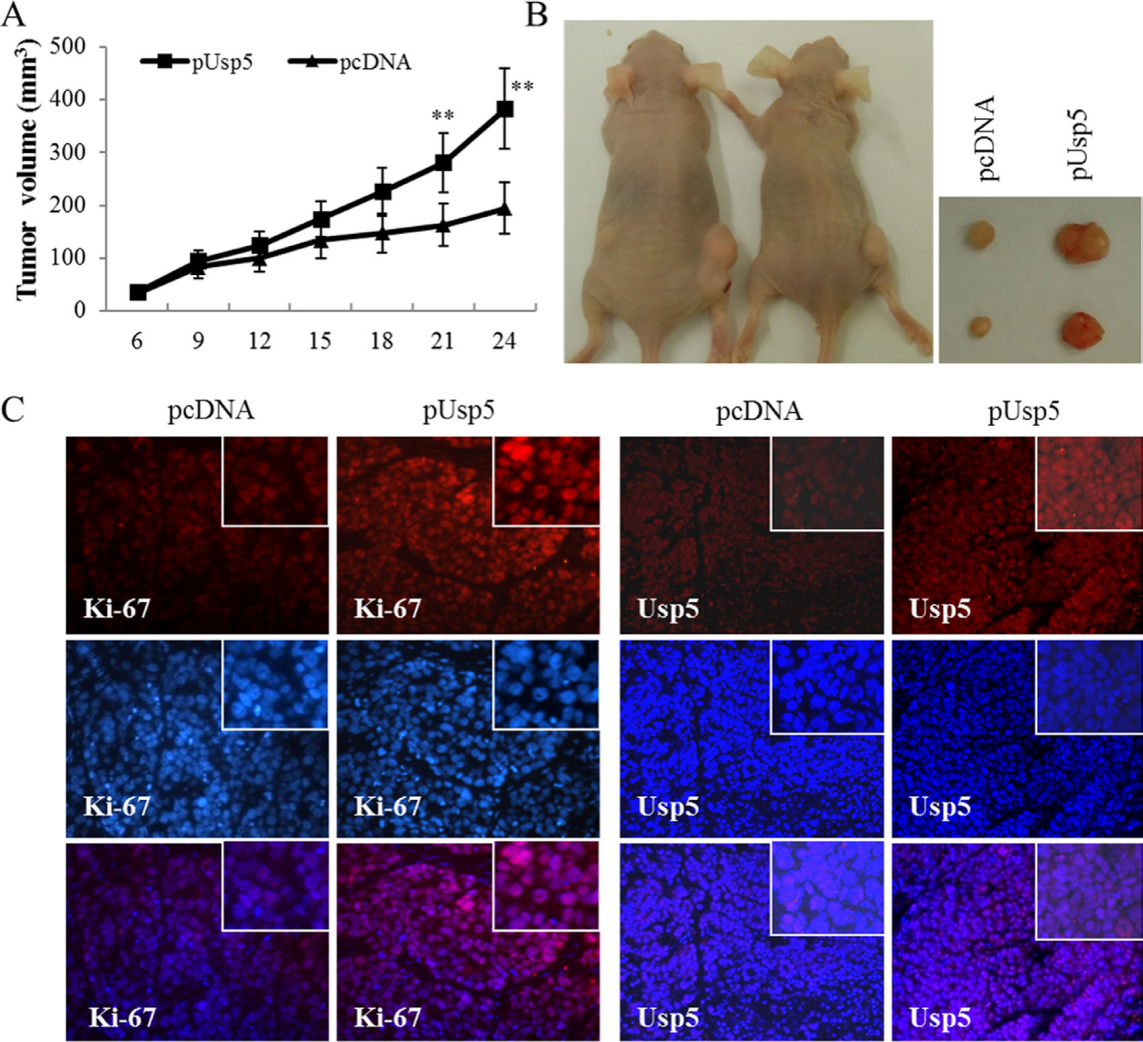
Usp5 overexpression promoted tumorigenicity *in vivo* HepG2 cells transfected with pUsp5 or pCDNA were injected *s.c*. into nude mice. (**A**) the growth curves of tumor volumes were measured. Each data point represented the mean ± SD of five mice. (**B**) pUsp5-transfected cells generated bigger tumors than their control cells. (**C**) the immunofluorescence of Ki-67 and Usp5 stained sections followed by counterstaining with DAPI. **P < 0.01.

On the other hand, an expression vector pCDNA-Usp5 (pUsp5), which encoded the full-length coding sequence of Usp5, was transfected into HepG2 cells (Figure 3A). Intriguingly, reinforced expression of Usp5 dramatically promoted cell viability (Figure 3B) and stimulated colony formation (Figure 3C). We further verified these *in vitro* findings by using an *in vivo* xenograft tumor model. The immortalized hepatic cell line LO2 stably transfected with pcDNA or pUsp5 were *s.c*. injected into the dorsal flank of nude mice. Compared with the control group, the pUsp5 group revealed a significant increase in tumor volumes (Figure 4A) and sizes (Figure 4B). Furthermore, the expression of cell proliferation marker Ki-67 and Usp5 were increased in xenografts of mice treated with pUsp5 cells by immunohistofluorescence analyses (Figure 4C).

### Usp5 promoted the migration and drug resistance in HCC cells

**Figure 5:**
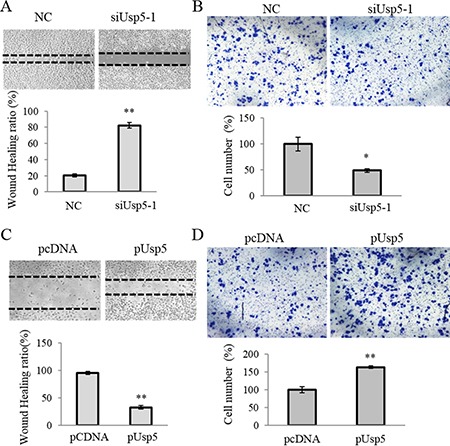
Usp5 stimulated migration of HCC cells (**A** and **B**) Usp5 knockdown suppressed the migration of HepG2 cells by using wound healing (A) and transwell analyses (B). (**C** and **D**) Usp5 overexpression stimulated the migration by using wound healing (C) and transwell analyses (D). **P < 0.01.

The cell migration capacity was examined and we found that Usp5 knockdown significantly suppressed the migrated cells by wound healing and transwell analyses (Figure 5A and 5B). Furthermore, more migrating cells were found in HepG2 cells with ectopic overexpression of Usp5 (Figure 5C and 5D).

**Figure 6:**
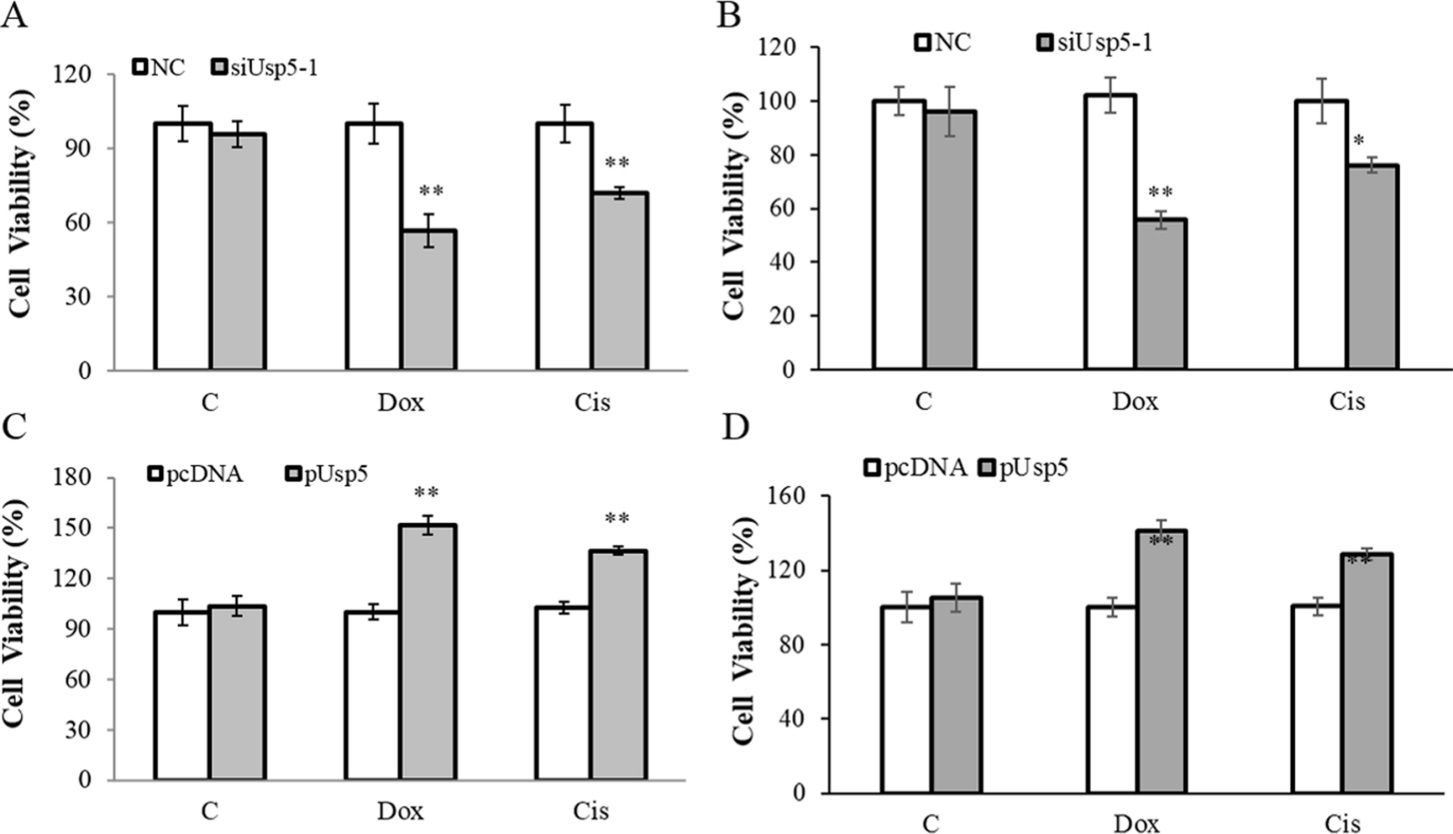
Usp5 promoted drug resistance in HepG2 cells (**A** and **B**) Usp5 knockdown suppressed the viabilities in HepG2 cells (A) and Bel7404 cells (B) with 2.0 μM Dox and 10 μM Cis treatment. (**C** and **D**) Usp5 overexpression promoted cell growth in the Dox and Cis treated HepG2 cells (C) and Bel7404 cells (D). **P < 0.01.

On the other hand, we also investigated the Usp5-regulated-drug resistance in HCC cells. The cells were transfected with siUsp5 or pUsp5, then treated with 2.0 μM Doxorubicin (Dox) and 10μM Cisplatin (Cis), which are two common anti-cancer chemotherapy drugs. Twenty-four hours later, the cell viabilities were measured. Intriguingly, with introduction of Dox or Cis, Usp5 knockdown suppressed (Figure 6A and 6B) while its overexpression promoted cell growth in the treated HepG2 cells and Bel7404 cells (Figure 6C and 6D), suggesting that Usp5 directs drug resistance in HCC cells.

### Usp5 knockdown induced the activation of p14^ARF^-p53 signaling in HCC cells

**Figure 7:**
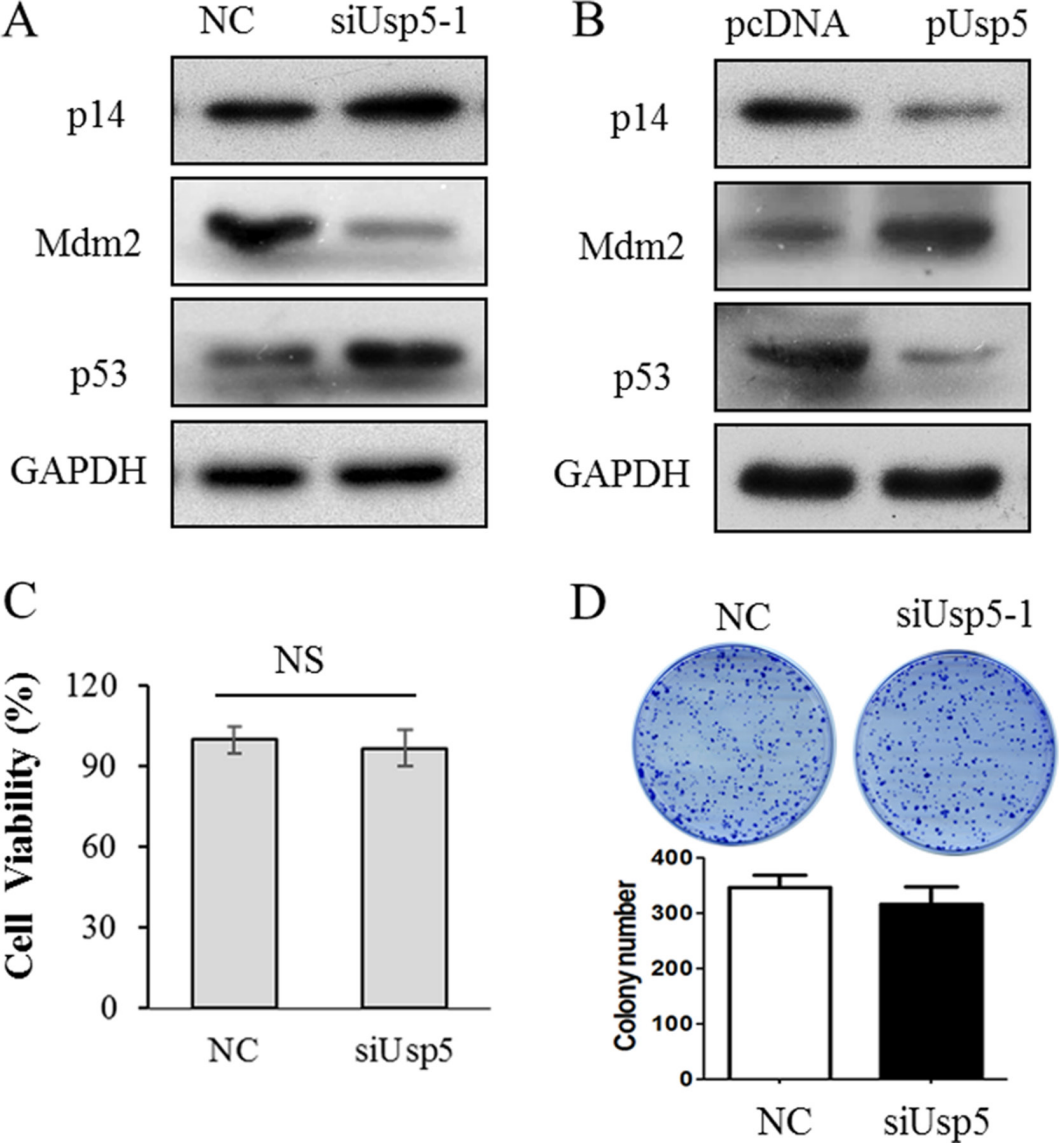
Inactivated p14^ARF^-p53 signaling was involved in Usp5 promoted tumorigenesis (**A**) p14^ARF^-p53 signaling was stimulated by siUsp5 including p14^ARF^ and P53 were upregulated, and Mdm2 was downregulated in HepG2 cells. (**B**) p14^ARF^ signaling was inactivated by pUsp5 including p14^ARF^ and p53 were downregulated, and Mdm2 was upregulated in HepG2 cells. (**C** and **D**) siUsp5-1 couldn't affect the cell viability (C) and colony formation (D) in Hep3B cells.

According to previous report, Usp5 reduced p53 transcriptional ability [17]. Therefore, we wondered whether p14^ARF^-p53 signaling was involved in the Usp5 mediated tumorigenicity of HCC. As expected, p14^ARF^ and p53 were significantly upregulated whereas Mdm2 was downregulated by siUsp5, indicating the activation of p14^ARF^-p53 signaling (Figure 7A). Conversely, Usp5 overexpression suppressed the expression of p14^ARF^ and p53 and promoted Mdm2 expression (Figure 7B), leading to inactivation of this signaling. To further confirm the Usp5 stimulated the tumorigenesis through p53, a p53-deleted HCC cells line Hep3B was transfected with siUSP5-1, the cell proliferation (Figure 7C) and colony formation (Figure 7D) all showed that they have no obvious difference compared with the NC group. All these data suggest that Usp5 promoted tumorigenesis *via* inactivation of p14^ARF^-p53 signaling in HCC cells.

## DISCUSSION

Ubiquitination is a critical regulator of most cellular pathways; therefore, elucidating the function of ubiquitination in tumorigenesis may provide insight for developing novel therapeutic targets. As a member of DUBs, Usp5 has been studied well, especially about their substrate specificity and kinetics [15–16, 20]. However, the role of Usp5 in tumorigenesis remains unknown. In this study, we firstly identified that Usp5 stimulated carcinogenesis in HCC as a novel player.

As a superfamily of DUBs, accumulating evidences demonstrate that Usp family are associated with carcinogenesis, such as Usp21 is significantly upregulated in cancer stem cells (CSCs) of renal cell carcinoma (RCC) cell lines, and it is considered as a novel diagnostic or therapeutic target for RCC [21]; Usp22 is upregulated in several malignancies in correlation to metastasis and poor survival [22–23], and acts as a poor prognostic factor in patients with non-small cell lung cancer (NSCLC), bladder cancer, cervical cancer, breast cancer, salivary duct carcinoma, and papillary thyroid carcinoma [24–26]. For Usp5, it has been demonstrated to play a significant role in glioblastoma [27] and melanoma [28]. In the present study, Usp5 was found to be upregulated in HCC cells and most clinical specimens. The siRNA-induced knockdown of Usp5 inhibited cell proliferation, migration ability and drug resistance. On the other hand, Usp5 overexpression promoted tumorigenesis and drug resistance. These data suggest that Usp5 plays a vital role in tumorigenesis of HCC.

p53 is a classical regulator in mediating cell proliferation and carcinogenesis [29]. This tumor suppressor plays important role in regulating cell cycle arrest and apoptosis. p14^ARF^ binds and inhibits the p53 antagonist Mdm2, leading to the accumulation of p53 [30]. Our study showed that Usp5 knockdown induced cell cycle arrest and apoptosis, at least partially associated with p53 expression. In previous study, p53 stabilization was mediated by Usp5 through accumulation of unanchored poly-ubiquitin chains which competed with p53 for entering into the proteasome [18]. This may also contribute to recruitment of Usp5 into the DNA damage as previously described [31]. Of note, the effect of Usp5 knockdown on p53 activity is distinct from that of an MDM2 inhibitor (MI219), which blocks p53 ubiquitination to circumvent its destruction [32]. Both Usp5 knockdown and MI219 alone lead to p53 increase and FAS induction, which were further elevated by co-treatment [32]. This suggests that Usp5 and MDM2 regulate p53 levels by distinct mechanisms. We therefore suppose that the p53 upregulation may be a combined effect of Usp5 knockdown and MDM2 repression in HCC. Usp5 inhibition could provide an alternate approach in reactivation of p53 in melanoma [33–34]. Our results showed that the p14^ARF^-p53 signaling was activated by Usp5 knockdown in HCC cells.

In conclusion, our study shows for the first time that Usp5 plays a critical role in hepatocarcinogenesis through inducing the inactivation of p14^ARF^-p53 signaling. As an oncogene, Usp5 contributes to tumorigenesis and drug resistance, which provides a potential therapeutic target to HCC.

## MATERIALS AND METHODS

### Cell culture and tissue specimens

A panel of HCC cell lines including HepG2, Bel7404, PLC5 and Huh7 as well as immortalized non-tumorigenic MIHA and LO2 cells were cultured in Dulbecco's modified Eagle's medium (DMEM, Invitrogen) supplemented with 10% FBS and 1% Penicillin-Streptomycin. Fifty paired primary HCC specimens and their non-tumor counterparts were collected by means of tumor resection at the Prince of Wales Hospital, The Chinese University of Hong Kong (CUHK). All the human tissues were obtained with informed consent, and this study was approved by Joint Chinese University of Hong Kong-New Territories Ease Cluster Clinical Research Ethics Committee (No. CRE-2012.215).

### RNA oligoribonucleotides and cell transfections

Two small interfering RNAs (siRNAs) that specifically target human Usp5 mRNA were purchased from Genepharma (Shanghai, China), were designated as siUsp5-1 and siUsp5-2. The negative control RNA duplex (NC) was non-homologous to any human genome sequences. Their sequences were listed as follows:

NC: 5′UUCUCCGAACGUGUCACGUUU3′;

siUsp5-1: 5′AACAGUAUGUGGAGAGACATT3′;

siUsp5-2: 5′ GCAGAUGGGUGAUCUACAATT3′.

RNA oligoribonucleotides transfection was performed by using Lipofectamine 2000 (Invitrogen), and all the experiments were repeated in triplicate.

### Usp5 overexpression plasmid construction and stable cell line establishment

The full coding sequence (CDS) of Usp5 was amplified and then subcloned into pCDNA3.1 vector. The Usp5 overexpression vector was designated as pUsp5, and the empty vector was used as control. LO2 cells were transfected with pUsp5 plasmids and positive pUsp5-transfected LO2 colonies were screened using G418 for several days. pUsp5 stable-expression LO2 cells were characterized using qRT-PCR assays.

### Cell viability, apoptosis and cell cycle analyses

Cell viability was analyzed by using 3-(4,5-Dimethylthiazol-2-yl)-2,5-diphenyltetrazolium bromide (MTT, Sigma) assays as described previously [35]. Briefly, LO2 cell were seeded at the density of 5 × 10^3^ cells per well into a 96-well plate. After miRNAs transfection, the cells were maintained for 72 hours and cell viabilities were determined by using a Benchmark Plus^TM^ microplate spectrometer (Bio-Rad). Tumor cell apoptosis was examined by using a FITC-labeled AnnexinV/propidium iodide (PI) Apoptosis Detection Kit (Invitrogen, CA). For cell cycle analysis, LO2 cells were seeded in 6-well plates at 2 × 10^5^ per well and transfected with miRNAs. After 72 hours, the cell cycle distribution was analyzed by propidium iodide (PI) staining cytometric assay [36].

### Colony formation assays

The pUsp5 plasmid or siUsp5 and their corresponding controls were transfected into HepG2 cells and cultured for 72 hours. The cells were then re-plated in 6-well plates at the density of 5 × 10^2^ per well and maintained for two weeks. The colonies were fixed and stained with 0.5% crystal violet for 15 minutes. The number of colonies was counted under a microscope.

### RNA extraction, reverse transcription and quantitative reverse-transcription polymerase chain reaction (qRT-PCR)

The total RNA was extracted by Trizol reagent (Invitrogen). The reverse transcription was performed as described previously [35]. Briefly, the total RNA was reversely transcribed by using ImProm-II^™^ Reverse Transcription System (Promega, WI). All the samples were applied to qRT-PCR analyses by using SYBR Green PCR master mix (Roche) on an ABI 7500 Real Time PCR System. Primers used for qRT-PCR were listed as follows: forward: 5′CGAGATGCCAGAGGAGCTCGACA3′; reverse: 5′AGCATGGGCGATGTCGGAGAG3′. GAPDH was used as an endogenous control.

### Western blotting

Protein lysates were separated by SDS-PAGE (10%) and transferred to PVDF membranes (Millipore). The membranes were blocked with 5% skimmed milk for 1 hour and incubated with primary antibodies including rabbit polyclonal anti-Usp5 (Cell Signaling Technology), anti-P14 (Santa Cruz), anti-Mdm2 (Santa Cruz), or anti-P53 (Cell Signaling Technology) at 4°C overnight. Then the membranes were incubated with HRP-labeled corresponding secondary antibodies for 1 hour and the chemiluminescence (ECL, USA) was used to detect the result. GAPDH was used as the internal control.

### Cell migration assays

The migration of cells was evaluated as described previously [37]. Briefly, the migration was evaluated by using wound healing and transwell assays. The transwell migration assays were performed in a Boyden chamber with 10-μm pore (Corning, USA). 1 × 10^5^ Usp5-overexpressing or knockdown HepG2 cells and their corresponding control cells were seeded into the upper compartment. After the desired incubation period, the non-migrated cells on the upper chamber were removed. Afterwards, the cells were fixed with 4% paraformaldehyde for 20 minutes and stained with 0.2% crystal violet (Sigma-Aldrich, USA) for 10 minutes. Images were captured and the data was analyzed by Image J software (National Institutes of Health, USA).

### Xenograft mouse model

Female athymic nude mice (4–6 weeks old) were purchased from the Laboratory Animal Services Centre of CUHK. The usage and treatment of nude mice were approved by the Animal Experimental Ethics Committee of CUHK. LO2 cells transfected with pUSP5 or pcDNA and 1 × 10^6^ transfected cells were injected *s. c*. into the dorsal flank of nude mice (*n* = 6). Tumor size was measured twice a week and tumor volumes (V) were calculated as the formula: V = (D × d^2^)/2, in which D means the longest diameter and d means the shortest diameter [38].

### Immunofluorescence

The specimens were fixed overnight in 4% paraformaldehyde, dehydrated and embedded in paraffin. Sections (5 μm) were used to analyze Ki-67 (Calbiochem) and Usp5 expression. After being counterstained with DAPI (Invitrogen), the images were captured using a Zeiss Axiophot 2 microscope.

### Statistical analysis

Data are expressed as means ± SD. The two-tailed Student's *t* test was used to compare cellular proliferation, cell cycle distribution, colony formation, gene expression, and tumorigenicity between the two selected groups. The correlation between two factors in HCC specimens was performed by using Pearson's correlation in GraphPad Prism 5.0. The difference was considered as statistically significant when the *P value* is less than 0.05.

## SUPPLEMENTARY MATERIALS FIGURE



## Abbreviations

CDS: the coding sequence
Cis: Cisplatin
CSCs: cancer stem cells
Dox: Doxorubicin
DUBs: deubiquitinating enzymes
HCC: hepatocellular carcinoma
MTT: 3-(4,5-Dimethylthiazol-2-yl)-2,5-diphenyltetrazolium bromide
NC: negative control RNA duplex
NSCLC: non-small cell lung cancer
PI: propidium iodide
pUsp5: the expression vector pCDNA-USP5
RCC: renal cell carcinoma
siRNAs: small interfering RNAs
siUsp5: specific siRNAs for Usp5
UPS: ubiquitin-proteasome system
Usp: ubiquitin specific protease.
