# Supplementary material for: Usp5 functions as an oncogene for stimulating tumorigenesis in hepatocellular carcinoma
Source: Oncotarget. 2017 Apr 6;8(31):50655–64. doi: 10.18632/oncotarget.16901 (PMC5584183; doi:10.18632/oncotarget.16901)
Supplement: Supplementary file 1 [file oncotarget-08-50655-s001.pdf]

Usp5 functions as an oncogene for stimulating tumorigenesis in hepatocellular carcinoma

Supplementary Materials

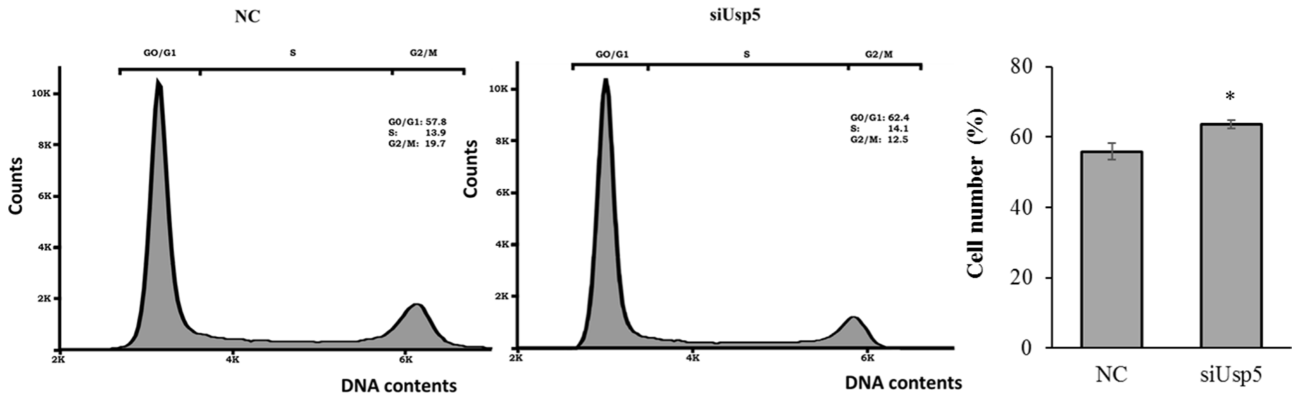

Supplementary Figure 1: The effect of siUsp5 on cell cycle in Be17404 cells. *$P < 0.05$.
